# Reevaluating the fraction of cancer attributable to excess weight: overcoming the hidden impact of prediagnostic weight loss

**DOI:** 10.1007/s10654-024-01146-0

**Published:** 2024-09-18

**Authors:** Fatemeh Safizadeh, Marko Mandic, Michael Hoffmeister, Hermann Brenner

**Affiliations:** 1 Division of Clinical Epidemiology and Aging Research, German Cancer Research Center, Im Neuenheimer Feld 581, D-69120 Heidelberg, Germany; 2 Medical Faculty Heidelberg, Heidelberg University, Heidelberg, Germany; 3 Division of Preventive Oncology, German Cancer Research Center (DKFZ) and National Center for Tumor Diseases (NCT), Heidelberg, Germany; 4 German Cancer Consortium (DKTK), German Cancer Research Center (DKFZ), Heidelberg, Germany

**Keywords:** Body mass index, Overweight, Obesity, Population attributable fraction, Cancer

## Abstract

**Objective:**

To evaluate the magnitude of the potential underestimation of the proportion of cancer cases attributable to excess weight, known as population attributable fraction (PAF), due to potential bias from prediagnostic weight loss already present at baseline of cohort studies and to overcome it as much as possible.

**Methods:**

Data from the UK Biobank cohort participants aged 40–69 without prior cancer diagnosis were analyzed. We assessed the magnitude of associations of excess weight with the incidence of obesity-related cancers combined, and separately for gastrointestinal (GI) and other cancers. Using multivariable Cox proportional hazards models, hazard ratios (HR) and their 95% confidence intervals (CI), and PAFs for excess weight at baseline were estimated for various periods of time after weight measurements.

**Findings:**

Of 458,660 participants, 20,218 individuals developed obesity-related cancers during a median 11.0-year follow-up, comprising 8,460 GI, and 11,765 non-GI cancers. PAFs were much higher for cancers occurring more than four years after recruitment than for cancers occurring within the initial four years: 17.7% versus 7.2%, 21.4% versus 11.7% for GI, non-GI and all obesity-related cancers combined, respectively. With respect to total cancer (including cancers with no established relationship with excess weight), PAFs were estimated as 5.1% and 8.8% for the 0–4 and 4-14-year periods of follow-up.

**Conclusion:**

The proportion of cancers attributable to excess weight is likely substantially larger than previously estimated based on cohort studies with short follow-up time or no or only limited exclusion of the early years of follow-up from the analyses.

**Supplementary Information:**

The online version contains supplementary material available at 10.1007/s10654-024-01146-0.

## Introduction

Overweight and obesity are well established risk factors for a variety of cancers, such as esophageal (adenocarcinoma), colorectal, pancreatic, renal, endometrial, and postmenopausal breast cancer [1, 2]. Based on meta-analyses of studies evaluating associations of excess weight with individual cancers for which evidence of a causal role was classified as sufficient or strong (either convincing or probable) by the International Agency for Research on Cancer (IARC) and the World Cancer Research Fund (WCRF), the total proportion of cancers attributable to excess weight has been estimated to be approximately 4% globally [3, 4] and in the order of 4 to 8% in different Western countries [5–11].

However, this proportion, which is commonly called population attributable fraction (PAF), may have been underestimated because prediagnostic weight loss may have led to underestimation of overweight and obesity related cancer risks in cohort studies, from which these risks have been derived. Cancer patients may suffer from substantial prediagnostic weight loss [12]. This particularly applies to patients with gastrointestinal (GI) cancers, with 75% of their cancer-related weight loss happening before diagnosis [13]. The tumor’s anatomical location affects physiological functions within the digestive system which can result in impaired nutrient absorption, malnutrition, and metabolic dysregulation, thereby accentuating the weight loss observed in such cases. As most cohort studies exclusively rely on weight measured at recruitment, associations between excess weight and cancer diagnoses may be substantially attenuated and potentially even reversed during the early years of follow-up, with potential attenuation persisting even in the longer run and being most salient in cohorts with relatively short follow-up periods. Even though sensitivity analyses excluding the initial year(s) of follow-up have been conducted by some cohort studies, such results were often not explicitly reported and included in meta-analyses, and even where this was the case, excluded periods of follow-up may have been too short to fully compensate potential underestimation of the association [14]. We have previously demonstrated for colorectal cancer how strongly relative risk estimates may vary according to follow-up time included in the analysis [15].

The aim of this study was to evaluate the impact of excess weight on cancer risk based on the UK Biobank cohort, paying particular attention to overcoming potential underestimation due to prediagnostic weight loss. We derived PAFs for the 13 cancer types for which a causal association with excess weight has been established, both overall, and separately for GI and non-GI cancers.

## Methods

### Study population and design

This study utilized data from the UK Biobank, a prospective cohort study comprising approximately 500,000 participants aged 40–69 years recruited between 2006 and 2010, from across the United Kingdom. Details of this study have been described elsewhere [16]. Extensive information on socio-demographic, lifestyle, and health-related factors was collected through a self-completed touch-screen questionnaire and computer-assisted interviews. In addition, physical and functional measurements were conducted, and data on cancer, death, and primary care were obtained through linkage to national cancer and death registries and electronic health records. Ethical approval for the UK Biobank was obtained from the North West Multi-centre Research Ethics Committee (MREC) as a Research Tissue Bank (RTB), the approval was renewed in 2021 (21/NW/0157), and all participants provided electronic signed informed consent. For this analysis, only participants with no previous cancer diagnosis (except non-melanoma skin cancer) and without missing body mass index (BMI) values at recruitment were included.

### Exposure ascertainment

Weight measurements were taken using the Tanita BC-418 MA body composition analyzer, and standing height was measured using a Seca 202 height measure during the initial assessment visit [17]. BMI was determined by dividing individuals’ weight in kilograms by the square of their height in meters. The World Health Organization (WHO) categories were used to classify BMI: <18.5 kg/m^2^ (underweight), ≥18.5-<25 kg/m^2^ (normal weight), ≥25-<30 kg/m^2^ (overweight), ≥30-<35 kg/m^2^ (obesity class I), ≥35-<40 kg/m^2^ (obesity class II), and ≥40 kg/m^2^ (obesity class III) [18].

### Cancer incidence

Information on cancer incidence was obtained from national cancer registries through linkage with the UK Biobank data. The International Statistical Classification of Diseases (ICD-10) was used to determine incident cancer cases. Thirteen cancer types with established causal association with excess weight according to IARC [1], which are listed in Supplemental Table 1, were included in the analyses. In this manuscript, these cancers are referred to as obesity-related cancers. Of these, GI cancers comprised cancers of the esophagus, stomach (cardia), colorectum, liver, gall bladder, and pancreas, while all other obesity-related cancers were categorized as non-GI cancers. The number of incident cancer cases by cancer type is also shown in Supplemental Table 1. This study includes complete cancer follow-up data until 29th of February 2020 for England and Wales and 31st of January 2021 for Scotland.

### Statistical analysis

All statistical analyses were conducted using SAS software version 9.4. Baseline characteristics of the cohort were summarized using descriptive statistics. The association between BMI and cancer risk was evaluated using multivariable Cox proportional hazards models. Follow-up time was defined as the time from initial assessment visit to the first cancer diagnosis, date of death, date of loss to follow-up, or the end of the follow-up period, whichever came first. Two models were fitted: the first model was adjusted for age at baseline (years, continuous) and sex (male, female), and the second (fully adjusted model) was adjusted for additional covariates including height (cm, continuous), self-reported ethnic background (classified as white, or other), socioeconomic status (Townsend deprivation index, continuous), educational qualifications (higher academic/professional, lower academic/vocational, or none), smoking status (never, former, current), pack-years of smoking (years, continuous), alcohol consumption (never, special occasions only, 1–3 times a month, once or twice a week, 3–4 times a week, daily or almost daily), level of physical activity determined by the International Physical Activity Questionnaire (IPAQ) [19] (low, moderate, high), fruit intake (pieces/day, continuous), vegetable intake (tablespoons/day, continuous), and red and processed meat intake (never, less than once a week, once a week, ≥ 2 a week), hormone replacement therapy (no, yes/women only), menopausal status (pre-menopausal, post-menopausal/women only), history of bowel cancer screening (no, yes), history of mammography (no, yes/women only), and family history of breast and colorectal cancer (no, yes). Schoenfeld residuals plots were examined to assess deviations from the proportionality assumption and none was detected. To address missing covariate values, the SAS multiple imputation procedure PROC MI was employed. The analysis involved combining five imputed datasets using PROC MIANALYZE. Specifically, physical activity data had a 20% rate of missing values, while all other covariates had less than 2% missing values and age and sex had no missing values.

Hazard ratios (HRs) and corresponding 95% confidence intervals (CIs) were calculated to quantify the cancer risk for each BMI category compared to the reference group of participants with normal BMI (18.5 kg/m^2^≤BMI<25 kg/m^2^). HRs (95% CIs) were computed for all obesity-related cancers, obesity-related GI cancers, and obesity-related non-GI cancers. Due to small numbers in the underweight category and obesity sub-categories, further analyses were conducted excluding the underweight participants (BMI<18.5 kg/m^2^) and using combined obesity sub-categories (≥30 kg/m^2^). PAFs (95% CIs) of cancer cases associated with excess weight (BMI≥25 kg/m^2^) for cancer incidence were then estimated based on the HRs calculated for the association between overweight and obesity with the various cancers and the prevalences of overweight and obesity in the population, using Mietinnen’s formula [20] modified for risk factors with multiple categories of exposure (Supplemental Text). Population prevalence of different BMI categories by sex- and 5-year age groups for years 2006–2010 (years of the UK Biobank recruitment) was extracted from a nationally representative survey, the “Health Survey for England – 2010” [21]. The data source is summarized in Supplemental Table 2.

PAFs were calculated as age- and sex-weighted averages accounting for the substantial variability in cancer incidence observed across different age and sex groups. Initially, age- (at baseline; 5-year increments) and sex-specific PAFs were calculated. The overall PAF was subsequently determined as a weighted mean of these specific PAFs, employing weights corresponding to the age- and sex-specific number of cancer cases. All PAFs were calculated as proportion of obesity-related cancer cases attributable to excess weight. Additionally, we calculated PAFs as proportion of total cancer cases (ICD-10 C00-C97 excluding C44) attributable to excess weight in our dataset (number of total cancer cases not shown).

To address the potential underestimation of calculated HRs and PAFs, the following analyses were performed (this approach has been detailed previously [15]): firstly, a standard cohort analysis was performed using the complete available follow-up period at the time of analysis (14 years). Subsequently, we performed separate analyses by including either only the initial four years of follow-up (0-4 years), or only the later years of follow-up (>4–14 years). The rationale for this analysis is as follows: During the early years of follow-up, it is likely that a significant proportion of newly diagnosed cancer cases originated from participants who already had preclinical cancer at the time of their recruitment, which may have led to some weight loss. For cancers diagnosed more than four years after diagnosis, relevant weight loss before recruitment would appear unlikely. We additionally did a sensitivity analysis by including 0–3/>3–14 and 0–5/>5–14 years of follow-up to assess the robustness of the findings from the main analysis.

All analyses were conducted for all obesity-related cancers combined, as well as separately for obesity-related GI and non-GI cancers.

Subgroup analyses were performed based on age group, sex, smoking status, and diabetes considering different follow-up time windows (0–4, >4–14, 0–14). All p-values reported in this study are two-sided, and statistical significance was defined as p-values less than 0.05.

## Results

**Fig. 1:**
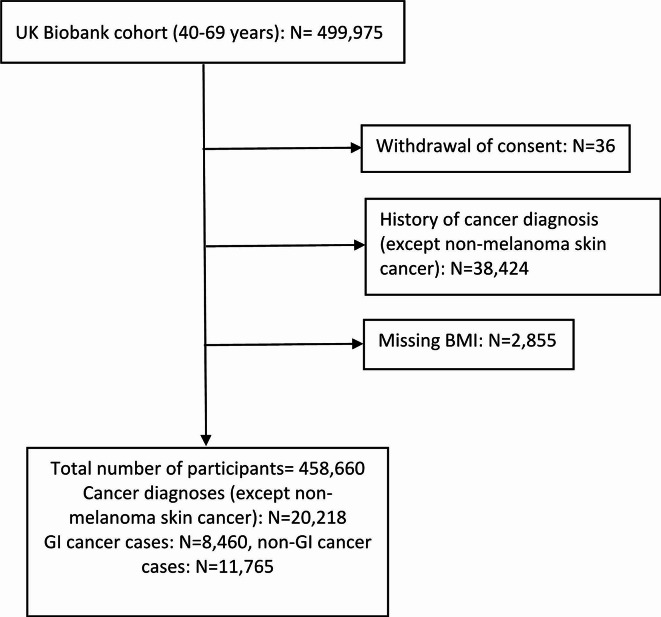
Study population flow diagram

The flow diagram representing the selection of the study population is shown in Fig. 1. Among the 499,975 participants aged 40–69 years, 36 individuals withdrew their consent, and 38,424 participants had a cancer diagnosis (except non-melanoma skin cancer) either at or prior to recruitment. Furthermore, 2,855 participants had missing values for BMI and were therefore excluded from the analyses. As a result, the final analysis included 458,660 participants, among whom 20,218 were diagnosed with obesity-related cancers during follow-up. Of these, 8,460 participants had a GI cancer and 11,765 had a non-GI cancer (including seven participants with both cancer types). Colorectal cancer accounted for 64% of all obesity-related GI cancers, while post-menopausal breast cancer represented 59% of non-GI obesity-related cancers (Supplemental Table 1). The median follow-up time was 11.0 (interquartile range: 10.2–11.7) years.

Table 1 presents the distribution of baseline characteristics of the study participants, and the median BMI and incidence of obesity-related cancers according to categories of those characteristics. 41.8% of the study population were older than 60 years old and the study population consisted of 46.7% male versus 53.3% female participants. The majority of the study population (94.5%) had a white ethnic background (British, Irish, or any other white background) and other ethnic backgrounds included mixed, Asian or Asian British, Black or Black British, Chinese, and other ethnic groups. Notably, participants with older age, male gender, non-white ethnic background, higher Townsend deprivation index, no educational qualification, history of smoking, higher frequency of alcohol consumption, and lower physical activity had higher median BMI. Moreover, individuals who reported consuming less than 2 portions of fruit and 3 portions of vegetables per day, or one or more times processed meat per week, also displayed higher median BMI values.

**Table 1:** Distribution of baseline characteristics of the study participants, and median body mass index (BMI) and incidence of obesity-related cancers according to categories of those characteristics

Characteristics	*N* (%)	BMI (kg/m^2^) Median (IQR)	*N* Cases	Person-years	Incidence rate per 1000 person-years
**Age at recruitment (years)**					
40–44	49,381 (10.8)	26.1 (23.5–29.4)	411	547,075	0.8
45–49	62,569 (13.6)	26.4 (23.7–29.6)	1,175	690,408	1.7
50–54	71,223 (15.5)	26.7 (24.0-30.1)	2,474	777,422	3.2
55–59	83,598 (18.2)	26.8 (24.2–30.1)	4,004	902,153	4.4
60–64	109,677 (23.9)	27.0 (24.4–30.0)	6,464	1,159,682	5.6
65–69	82,212 (17.9)	27.1 (24.6–30.0)	5,690	852,382	6.7
**Sex**					
Male	214,236 (46.7)	27.3 (25.0-30.1)	6,594	2,302,439	2.9
Female	244,424 (53.3)	26.1 (23.4–29.7)	13,624	2,626,683	5.2
**Race/ethnicity**					
White	431,218 (94.5)	26.7 (24.1–29.9)	19,398	4,640,863	4.2
Other	25,237 (5.5)	27.1 (24.4–30.4)	728	265,329	2.7
**Deprivation Index (quartiles)**					
1 (Most affluent)	114,512 (25.0)	26.4 (24.0-29.3)	5,100	1,241,947	4.1
2	114,513 (25.0)	26.6 (24.1–29.6)	5,222	1,234,459	4.2
3	114,547 (25.0)	26.8 (24.2–30.0)	4,982	1,227,542	4.1
4 (Most deprived)	114,524 (25.0)	27.3 (24.4–30.9)	4,898	1,219,063	4.0
**Educational Qualifications**					
Higher academic/professional	224,534 (49.5)	26.2 (23.7–29.2)	9,156	2,425,579	3.8
Lower academic/vocational	152,081 (33.6)	27.1 (24.5–30.3)	6,454	1,633,551	4.0
None	76,616 (16.9)	27.8 (25.1–31.1)	4,354	812,742	5.4
**Smoking status**					
Never	251,788 (55.2)	26.4 (23.9–29.6)	10,289	2,725,875	3.8
former	156,237 (34.2)	27.3 (24.7–30.4)	7,729	1,667,102	4.6
Current	48,354 (10.6)	26.5 (23.8–29.6)	2,106	512,447	4.1
**Pack-years of smoking (years)**					
0	252,880 (65.2)	26.0 (23.3–29.6)	10,353	2,737,084	3.8
>0–20	71,271 (18.4)	26.0 (23.4–29.5)	3,102	765,161	4.1
>20–40	43,916 (11.3)	27.2 (24.3–30.9)	2,296	465,568	4.9
>40	19,905 (5.1)	28.2 (25.0-32.1)	1,336	202,483	6.6
**Alcohol consumption**					
Never	93,193 (20.4)	26.3 (23.9–29.0)	4,281	997,704	4.3
Special occasions only	106,436 (23.3)	26.4 (24.0-29.3)	4,312	1,151,248	3.7
One to three times a month	118,392 (25.9)	26.8 (24.3–30.0)	4,908	1,279,185	3.8
Once or twice a week	50,995 (11.1)	27.2 (24.4–30.8)	2,191	549,141	4.0
Three or four times a week	52,140 (11.4)	27.6 (24.5–31.6)	2,689	555,118	4.8
Daily or almost daily	36,481 (8.0)	27.4 (24.3–31.2)	1,790	386,175	4.6
**Physical activity (IPAQ groups)**					
Low	69,096 (18.8)	27.8 (24.9–31.3)	3,158	739,303	4.3
Moderate	150,039 (40.7)	26.6 (24.1–29.7)	6,673	1,612,097	4.1
High	149,402 (40.5)	26.2 (23.8–29.1)	5,900	1,606,682	3.7
**Fruit intake (pieces/day)**					
<2	127,108 (27.8)	27.1 (24.4–30.2)	5,112	1,362,244	3.8
≥2-<5	241,214 (52.8)	26.7 (24.1–29.9)	10,825	2,596,526	4.2
≥5	88,725 (19.4)	26.3 (23.8–29.6)	4,217	953,680	4.4
**Vegetable intake (tablespoons/day)**					
<3	82,742 (18.2)	27.0 (24.3–30.2)	3,356	893,000	3.8
≥3-<6	230,271 (50.6)	26.6 (24.1–29.7)	10,303	2,478,671	4.2
≥6	141,873 (31.2)	26.8 (24.2–30.0)	6,405	1,517,754	4.2
**Red meat intake**					
Never	30,685 (6.8)	25.1 (22.6–28.1)	1,210	330,502	3.7
Less than once a week	154,438 (34.1)	27.3 (24.6–30.5)	7,157	1,658,859	4.3
Once a week	98,262 (21.7)	26.8 (24.3–29.9)	4,343	1,060,183	4.1
≥2 times a week	170,134 (37.5)	26.5 (24.0-29.6)	7,278	1,825,506	4.0
**Processed meat intake**					
Never	42,272 (9.3)	25.2 (22.8–28.3)	1,807	455,553	4.0
Less than once a week	138,244 (30.3)	26.4 (23.8–29.5)	6,616	1,489,601	4.4
Once a week	133,427 (29.2)	26.9 (24.4–30.0)	5,917	1,435,042	4.1
≥2 times a week	143,021 (31.3)	27.4 (24.8–30.6)	5,805	1,531,374	3.8
**Family history of cancer**					
No	358,452 (79.8)	26.7 (24.1–29.9)	14,883	3,859,140	3.9
Yes	90,750 (20.2)	26.8 (24.2–29.9)	4,938	969,859	5.1
**Diabetes**					
Non-diabetic	432,963 (94.8)	26.0 (23.4–29.5)	18,645	4,664,653	4.0
Diabetic	23,684 (5.2)	31.2 (27.2–36.0)	1,486	243,577	6.1
**History of bowel cancer screening**					
No	313,025 (69.5)	26.7 (24.1–29.9)	12,638	3,407,313	3.7
Yes	137,139 (30.5)	26.9 (24.3–30.0)	7,267	1,430,713	5.1
**History of mammography (women only)**					
No	51,908 (21.3)	25.6 (22.9–29.4)	1,118	573,092	2.0
Yes	191,767 (78.7)	26.2 (23.6–29.8)	12,473	2,045,731	6.1
**Hormone replacement therapy (women only)**					
No	151,564 (62.3)	25.9 (23.2–29.6)	7,132	1,638,126	4.4
Yes	91,596 (37.7)	26.5 (23.8–29.9)	6,420	975,325	6.6
**Menopausal status (women only)**					
Pre-menopausal	60,519 (26.6)	25.3 (22.7–29.0)	1,400	667,439	2.1
Post-menopausal	167,213 (73.4)	26.3 (23.7–29.8)	11,660	1,776,478	6.6

*The total number of participants might not add up to 458,660 for some covariates due to missing data. The percentages might not add up to 100 due to rounding

Abbreviations: *BMI* Body Mass Index; *IPAQ* International Physical Activity Questionnaire; *IQR* Interquartile Range

The association between different BMI categories and the risk of all obesity-related cancers and obesity-related GI and non-GI cancers, is displayed in Table 2. The results are shown for age and sex adjusted and fully adjusted models, and given the high similarity in the results, only the findings from the fully adjusted model are presented for further analyses. The HRs (95% CIs) for obesity-related cancer risk demonstrated a monotonic increase with higher BMI values, with 1.18 (1.14–1.22) for overweight to 1.94 (1.78–2.11) for obesity class III, respectively, compared to individuals with normal BMI. A statistically non-significant lower risk was observed for the small group of underweight individuals. Obesity-related non-GI cancers exhibited a stronger association with increased BMI compared to obesity-related GI cancers, with hazard ratios for non-GI and GI cancers of 1.20 (1.15–1.26) and 1.14 (1.08–1.21) for overweight, which increased to 2.08 (1.88–2.31) and 1.71 (1.48–1.98) for obesity class III, respectively.

**Table 2:** Hazard ratios (95% CIs) for the incidence of overall obesity-related cancers, and obesity-related GI and non-GI cancers, according to BMI categories for complete follow-up years

Characteristic					
	*N* participants	Person-years	*N* cases	HR (95% CI)	HR (95% CI)
**Overall obesity-related cancers**	458,660	4,929,121	20,218	Age- and sex- adjusted model	Fully adjusted model^a^
**BMI at baseline (kg/m** ^**2**^ **)**					
<18.5 (underweight)	2,354	24,770	79	0.85 (0.68–1.06)	0.82 (0.66–1.03)
≥18.5-<25 (normal weight)	148,756	1,606,631	5,660	Ref.	Ref.
≥25-<30 (overweight)	195,321	2,101,887	8,423	1.16 (1.12–1.20)	1.18 (1.14–1.22)
≥30-<35 (obesity class I)	80,441	860,014	4,017	1.34 (1.29–1.40)	1.34 (1.29–1.41)
≥35-<40 (obesity class II)	22,894	242,630	1,409	1.62 (1.53–1.72)	1.62 (1.52–1.72)
≥40 (obesity class III)	8,894	93,188	630	1.95 (1.80–2.12)	1.94 (1.78–2.11)
**Obesity-related GI cancers**	458,660	4,984,404	8,460		
**BMI at baseline (kg/m** ^**2**^ **)**					
<18.5 (underweight)	2,354	25,020	28	0.99 (0.68–1.44)	0.89 (0.61–1.29)
≥18.5-<25 (normal weight)	148,756	1,623,385	2,122	Ref.	Ref.
≥25-<30 (overweight)	195,321	2,123,680	3,797	1.14 (1.08–1.20)	1.14 (1.08–1.21)
≥30-<35 (obesity class I)	80,441	870,375	1,769	1.31 (1.23–1.40)	1.27 (1.19–1.35)
≥35-<40 (obesity class II)	22,894	246,716	532	1.53 (1.40–1.69)	1.45 (1.31–1.60)
≥40 (obesity class III)	8,894	95,228	212	1.84 (1.60–2.12)	1.71 (1.48–1.98)
**Obesity-related non-GI cancers**	458,660	4,955,842	11,765		
**BMI at baseline (kg/m** ^**2**^ **)**					
<18.5 (underweight)	2,354	24,854	51	0.79 (0.60–1.05)	0.78 (0.59–1.03)
≥18.5-<25 (normal weight)	148,756	1,613,608	3,539	Ref.	Ref.
≥25-<30 (overweight)	195,321	2,114,201	4,630	1.18 (1.13–1.23)	1.20 (1.15–1.26)
≥30-<35 (obesity class I)	80,441	865,354	2,250	1.36 (1.29–1.44)	1.40 (1.32–1.48)
≥35-<40 (obesity class II)	22,894	244,128	877	1.68 (1.56–1.81)	1.74 (1.61–1.88)
≥40 (obesity class III)	8,894	93,698	418	2.00 (1.81–2.21)	2.08 (1.88–2.31)

^a^Adjusted for age, sex, height, ethnicity, socio-economic deprivation, education, smoking status, pack-years of smoking, alcohol consumption, physical activity, fruit, vegetable, red meat and processed meat intake, hormone replacement therapy (women only), menopausal status (women only), history of bowel cancer screening, history of mammography (women only), and family history of breast and colorectal cancer

Abbreviations: *BMI* Body Mass index; *CI* Confidence Interval; *GI* Gastrointestinal; *HR* Hazard Ratio; *Ref* Reference

Table 3 presents the hazard ratios (95% CIs) for the association between overweight and obesity with the risk of all obesity-related cancers and obesity-related GI and non-GI cancers, including various follow-up periods in the analysis. Inclusion of a short follow-up period (four years) in the analysis resulted in weak associations, and substantially stronger associations were observed for later follow-up years. For instance, overweight and obesity were associated with 1.11 (1.05–1.18) and 1.29 (1.20–1.38) fold increased risk of all obesity-related cancers within the first 4 years of follow-up, while they were associated with 1.21 (1.16–1.26) and 1.51 (1.44–1.59) fold increased risk, respectively, after 4 years of follow-up. The key patterns observed were replicated in the sensitivity analysis (Supplemental Table 3) with weaker associations when only 3 and 5 years of follow-up were included in the analysis, and much stronger associations when the first 3 and 5 years of follow-up were excluded from the analysis.

**Table 3:** Hazard ratios (95% CIs) for the incidence of overall, GI, and non-GI obesity-related cancers associated with overweight and obesity, obtained with inclusion of various follow-up time windows after recruitment in the analyses

Included follow-up years	*N* cases	HR^a^ (95% CI)	
		Overweight	Obesity
**Overall obesity-related cancers**			
**0–4**	6,213	1.11 (1.05–1.18)	1.29 (1.20–1.38)
**>4–14**	13,926	1.21 (1.16–1.26)	1.51 (1.44–1.59)
**0–14 (complete follow-up)**	20,139	1.18 (1.14–1.22)	1.44 (1.38–1.49)
**Obesity-related GI cancers**			
**0–4**	2,487	1.07 (0.97–1.18)	1.13 (1.01–1.26)
**>4–14**	5,945	1.17 (1.10–1.25)	1.41 (1.31–1.52)
**0–14 (complete follow-up)**	8,432	1.14 (1.08–1.21)	1.31 (1.23–1.39)
**Obesity-related non- GI cancers**			
**0–4**	3,728	1.14 (1.05–1.23)	1.39 (1.27–1.52)
**>4–14**	7,986	1.23 (1.16–1.30)	1.59 (1.50–1.69)
**0–14 (complete follow-up)**	11,714	1.20 (1.15–1.26)	1.52 (1.45–1.60)

^a^Adjusted for age, sex, height, ethnicity, socio-economic deprivation, education, smoking status, pack-years of smoking, alcohol consumption, physical activity, fruit, vegetable, red meat and processed meat intake, hormone replacement therapy (women only), menopausal status (women only), history of bowel cancer screening, history of mammography (women only), and family history of breast and colorectal cancer

Abbreviations: *BMI* Body Mass index; *CI* Confidence Interval; *GI* Gastrointestinal; *HR* Hazard Ratio

Table 4 includes the results of the sub-group analyses for obesity-related cancer risk, which reveal similar patterns to those observed in the main analyses. In summary, the HRs (95% CIs) were consistently lower for cancers diagnosed in the initial four years of follow-up than for later diagnosed cancers. However, this difference was much larger for males versus females, diabetics versus non-diabetics and particularly salient for current smokers, for whom no association of overweight or obesity with cancers diagnosed in the initial four years was observed, in contrast to particularly strong associations with cancers diagnosed in later years.

**Table 4:** Subgroup-specific hazard ratios (95% CIs) for obesity-related cancer incidence associated with overweight and obesity, obtained with inclusion of various follow-up time windows after recruitment in the analyses

Subgroup	Follow-up years	*N* cases	HR^a^ (95% CI)	
			Overweight	Obesity
**Age**				
<60	0–4	2,199	1.14 (1.03–1.26)	1.28 (1.14–1.44)
	>4–14	5,837	1.22 (1.15–1.33)	1.53 (1.43–1.64)
	0–14	8,036	1.20 (1.13–1.27)	1.46 (1.38–1.55)
≥60	0–4	4,014	1.11 (1.02–1.30)	1.28 (1.17–1.39)
	>4–14	8,089	1.22 (1.15–1.29)	1.50 (1.41–1.59)
	0–14	12,103	1.18 (1.13–1.24)	1.42 (1.36–1.50)
**Sex**				
Male	0–4	1,914	1.09 (0.97–1.23)	1.24 (1.08–1.41)
	>4–14	4,668	1.26 (1.17–1.37)	1.64 (1.50–1.79)
	0–14	6,582	1.21 (1.13–1.29)	1.51 (1.40–1.62)
Female	0–4	4,299	1.12 (1.04–1.20)	1.31 (1.20–1.42)
	>4–14	9,258	1.19 (1.13–1.25)	1.46 (1.38–1.55)
	0–14	13,557	1.17 (1.12–1.21)	1.41 (1.35–1.48)
**Smoking status**				
Non-smoker	0–4	3,136	1.11 (1.02–1.20)	1.37 (1.24–1.50)
	>4–14	7,112	1.16 (1.10–1.23)	1.51 (1.42–1.61)
	0–14	10,248	1.15 (1.10–1.21)	1.47 (1.39–1.55)
Former smoker	0–4	2,425	1.16 (1.04–1.28)	1.24 (1.11–1.40)
	>4–14	5,285	1.22 (1.13–1.31)	1.49 (1.37–1.61)
	0–14	7,710	1.20 (1.13–1.28)	1.40 (1.31–1.50)
Current smoker	0–4	609	0.94 (0.78–1.13)	1.06 (0.85–1.32)
	>4–14	1,479	1.39 (1.23–1.58)	1.51 (1.33–1.73)
	0–14	2,088	1.23 (1.11–1.37)	1.43 (1.27–1.61)
**Diabetes**				
No	0–4	5,709	1.11 (1.04–1.18)	1.25 (1.16–1.34)
	>4–14	12,858	1.20 (1.14–1.25)	1.47 (1.41–1.55)
	0–14	18,567	1.17 (1.13–1.21)	1.40 (1.35–1.47)
Yes	0–4	475	1.08 (0.77–1.51)	1.21 (0.88–1.67)
	>4–14	1,011	1.47 (1.13–1.91)	1.79 (1.39–2.31)
	0–14	1,486	1.31 (1.07–1.61)	1.58 (1.30–1.93)

^a^Adjusted for age, sex, height, ethnicity, socio-economic deprivation, education, smoking status, pack-years of smoking, alcohol consumption, physical activity, fruit, vegetable, red meat and processed meat intake, hormone replacement therapy (women only), menopausal status (women only), history of bowel cancer screening, history of mammography (women only), and family history of breast and colorectal cancer

Abbreviations: *BMI* Body Mass Index; *CI* Confidence Interval; *HR* Hazard Ratio

The results of the sub-group analyses for obesity-related GI and non-GI cancers are presented in Supplemental Tables 4 and 5, respectively, and followed the same trends as for overall obesity-related cancer risk considering various periods of follow-up time. For GI cancers, all associations were substantially stronger for men than for women and among diabetics and current smokers.

**Fig. 2:**
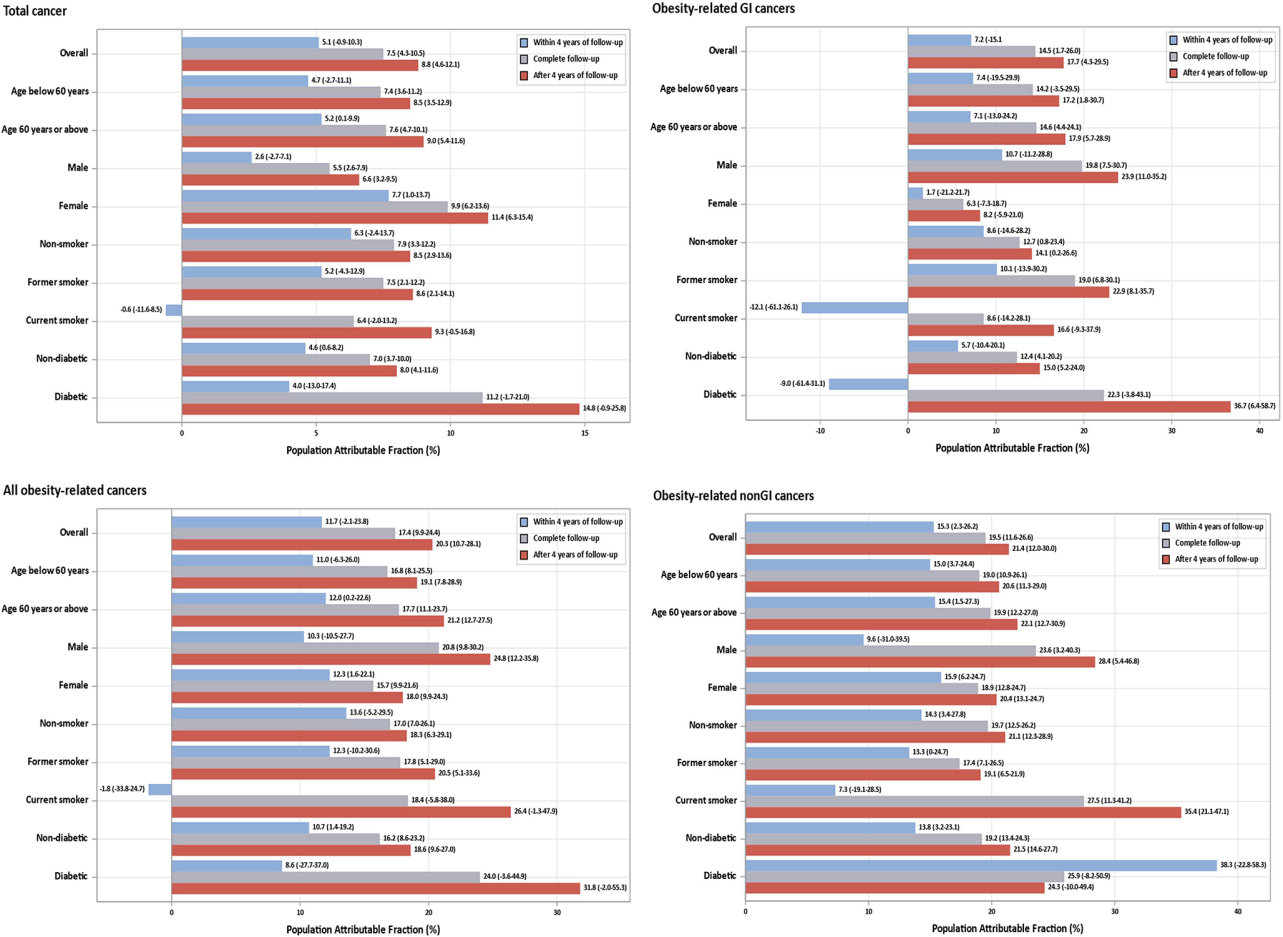
Percentage of total cancer, all, gastrointestinal (GI), and non-GI obesity-related cancers attributable to overweight and obesity with including 0–4, 0–14 (complete), and >4–14 years of follow-up

PAFs (95% CI) of total cancer, overall, GI and non-GI obesity-related cancers for overweight and obesity calculated in main and subgroup analyses within the four initial years of follow-up, complete follow-up, and after four years of follow-up are illustrated in Fig. 2. PAFs of cancer cases associated with overweight and obesity followed the same pattern, were small when only the initial follow-up years were included, and substantially increased for cancers diagnosed in later years of follow-up. For total cancer (including cancers for which an association with overweight or obesity has not been established), PAF estimates were 5.1% (-0.9-10.3%) in the analysis including only the initial four years of follow-up, and 8.8% (4.6-12.1%) for cancers diagnosed more than 4 years after recruitment. The PAF was estimated as 7.5% (4.3-10.5%) with including complete years of follow-up.

When PAFs were calculated as proportion of obesity-related cancers attributable to excess weight (and not as a proportion to total cancer incidence), PAFs were 11.7% (-2.1-23.8%) and 20.3% (10.7-28.1%) for cancers diagnosed in the first four years and in later years of follow-up, respectively. PAFs were higher for non-GI cancers than for GI cancers, but the difference in PAFs between cancers diagnosed in earlier and later years of follow-up was substantially higher for GI-cancers (7.2% versus 17.7%) than for non-GI cancers (15.3% versus 21.4%).

As for the HRs, patterns were similar in the sensitivity analyses (Supplemental Table 3). Proportions of cancers attributable to excess weight were 4.3% for cancers diagnosed within the first 3 years, 6.1% for those diagnosed within the first 5 years, and 8.4% for cancers diagnosed both more than 3 and 5 years after recruitment.

## Discussion

In our study, we aimed to reassess the association between excess weight and cancer risk and the proportion of cancer cases attributable to overweight and obesity (PAF) taking potential bias introduced by prediagnostic weight loss into account, a factor often overlooked in previous research. Our findings revealed that when accounting for bias induced by prediagnostic weight loss, the association was substantially stronger and the PAFs for obesity-related cancer incidence were considerably higher than when this source of bias is not addressed. These results suggest that PAF estimates of overall cancer cases associated with excess weight might markedly surpass previously available estimates.

As described, we demonstrated the weak excess weight-cancer risk associations over the initial years of follow-up (3–5 years) during which most of the diagnosed cancers are likely to have been in the preclinical phase already at recruitment. The potential underestimation in risk estimates and PAFs of cancer cases associated with excess weight due to prediagnostic weight loss was then addressed by excluding this time which was based on the average duration of preclinical yet detectable phase (preclinical sojourn time) of different cancers included in our analyses [22–25]. The difference between earlier and later years was most salient in current smokers among whom no association between excess weight and cancer incidence was observed during the first four years of follow-up and contrarily, a very strong association emerged with cancers diagnosed more than four years after recruitment. This difference was also substantially larger for men compared to women which might be due to higher prevalence of cancer cachexia, greater weight loss and muscle wasting among men with cancer versus women [26].

We also examined obesity-related GI and non-GI cancers separately. Consistent with the findings for overall obesity-related cancer risk, accounting for potential bias from prediagnostic weight loss revealed higher risk estimates and PAFs especially for GI cancers. For the GI cancers diagnosed 4–14 years after recruitment, 17.7% were estimated to be due to overweight and obesity, compared to only 7.2% of cancers diagnosed within four years. PAFs associated with excess weight were as high as 23.9% among men and 36.7% in diabetics compared to 8.2% in women and 15.0% in non-diabetics for GI cancers diagnosed more than 4 years after recruitment. These estimates highlight the potential underestimation of the excess weight-cancer risk association in epidemiological studies, particularly those involving GI cancers, when prediagnostic weight loss bias is not properly addressed, either due to a short follow-up or insufficient exclusion of initial follow-up years. It is noteworthy that the underlying mechanisms for sex differences regarding the association between excess weight and obesity-related GI cancers are not fully understood, but can be partly explained by higher visceral adipose deposition in men, hormonal factors such as protective effect of estrogens in women, and genetic differences [27, 28]. Moreover, gluconeogenesis and insulin resistance are featuring metabolic alterations in diabetes that might contribute to muscle loss and pre-existing diabetes has been shown to increase weight loss and aggravate cachexia in pancreatic and colorectal cancer patients [29, 30].

Unintentional weight loss prior to cancer diagnosis is a frequently observed symptom across various cancer types [12, 31, 32]. Moreover, particularly strong associations have been shown between unintentional weight loss and diagnoses of cancers primarily affecting the digestive system, such as gastro-esophageal, pancreatic, and colorectal cancers [33]. The primary factors underlying unexpected weight loss in cancer patients, known as cancer cachexia, include anorexia or decreased food intake, as well as increased catabolism resulting from tumor metabolism [34] which can be more pronounced in GI cancers due to the anatomical location of the tumor [13]. Cancer cachexia is characterized by the loss of skeletal muscle mass, with or without concurrent loss of fat mass. Due to prediagnostic weight loss, BMI measurements obtained in epidemiological studies, usually at a single time point near diagnosis (case-control studies) or study enrollment (cohort studies), may underestimate patients’ typical BMI. Consequently, risk estimates for the association between BMI and cancer and PAFs calculated based on these risk estimates may be underestimated.

In a previous UK–wide study, Brown et al. estimated that overweight and obesity accounted for 6.3% (men: 5.2%, women: 7.5%) of all reported cancer cases in the UK in 2015 [9], constituting the second-largest avoidable cause of cancer. Relative risks were extracted preferably from meta-analyses and cohort studies. Prevalence of excess weight and cancer incidence data were obtained from nationally representative population surveys and national data releases, respectively. Even without consideration of prediagnostic weight loss (i.e., when including the early years of follow-up of the UK Biobank cohort in the analysis) we obtained a somewhat higher overall PAF estimate (7.5%; men: 5.5%, women: 9.9%) which may be explained in part by the somewhat higher prevalence of overweight and obesity in the UK general population in the years 2006–2010 used for our analyses compared to 2005 in Brown et al. study. Additional consideration of prediagnostic weight loss by excluding the initial four years of follow-up further increased the total PAF estimate to 8.8% (men: 6.6%, women: 11.4%), suggesting that excess weight accounts for a substantially larger share of cancers than previously thought.

A study evaluating the proportion of cancer cases attributable to modifiable risk factors in the United States estimated that 7.8% (men: 4.8%, women:10.9%) of cancer cases in 2014 were attributable to excess weight [11]. The higher PAF estimates compared to the study of Brown et al. for the UK for 2015 may primarily reflect the much higher prevalence of obesity in the United States. This prevalence have further increased in recent years, with 30.7% and 42.4% of adults being overweight and obese in the US in 2017–2018 [35], and 37.9% and 25.9% of adults being overweight and obese in the UK in 2021 [36], respectively. It is plausible to assume that due consideration of prediagnostic weight loss would likewise have led to higher PAF estimates for the US in 2014, and that these PAFs further increased in the meantime due to the ongoing obesity epidemic in the past decades.

Associations of weight loss with increased subsequent cancer risk are well established. For example, in a retrospective study of 43,302 primary care patients with repeated weight measurements from the US, adjusted hazard ratios (95% CIs) for any cancer diagnosis with up to 9 years of follow-up ranged from 1.04 (1.02 to 1.05, *P* <0.001) for 1% weight loss to 1.44 (1.23 to 1.68, *P* <0.001) for 10% among 60 years old people [33]. In an own large population-based case-control study from Germany [37], we even observed a more than 7-fold increased risk (adjusted odds ratio, 7.5; 95% CI, 5.6–10.1) of colorectal cancer among participants who had lost more than 2 kg of weight during the preceding 2 years.

### Strengths and limitations

The large number of UK biobank participants and therefore, incident cancer cases, which enabled us to perform analyses including various periods of follow-up time, yet maintaining statistical power, is a major strength of our study. Furthermore, the BMI reported in the UK Biobank is based on measured weight and height using highly standardized methods compared to the self-reported BMI in other studies and hence not affected by reporting bias. Lastly, the comprehensive database available made it possible to account for a wide range of confounders in our models.

Although we aimed to minimize the underestimation of PAFs of cancer cases associated with excess weight, our study also has some limitations which in turn may have led to an underestimation of the calculated PAFs. First, the database and our analysis were restricted to one-time BMI measures at baseline. Higher PAFs would be expected, if information on life-time exposure to excess weight had been available, as suggested by a large population-based study from Germany [38]. In addition, “true PAFs for excess weight” would be even substantially higher when using more informative metrics of central obesity, which have been shown to be stronger predictors of a number of obesity-related cancers such as colorectal [39, 40], and pancreatic cancers [41, 42], compared to BMI. Furthermore, residual confounding cannot be ruled out, despite including a broad range of covariates in our models. Moreover, applying a uniform preclinical sojourn time cut-off (3–5 years), which represents an average across various cancer types, may not fully account for inherent heterogeneity of cancers, with varying preclinical periods. Finally, the predominantly white ethnicity of the UK Biobank participants limits the generalizability of our findings to other populations.

## Conclusions

In conclusion, our study suggests that the proportion of cancers attributable to overweight and obesity may be substantially larger than suggested by previous studies that paid no or less comprehensive attention to potential underestimation of associations by prediagnostic weight loss. In light of the steadily increasing prevalence of overweight and obesity in many parts of the world and the adverse health consequences of these conditions for many other common diseases, our results underline the need for enhanced efforts to cope with the ongoing obesity epidemic to limit the burden of cancer and other major diseases in the years to come.

## Electronic supplementary material

Below is the link to the electronic supplementary material.

Supplementary Material 1

## Data Availability

Data was re-used with the permission of the UK Biobank. This work used data provided by patients and collected by the NHS as part of their care and support. The UK Biobank is an open-access resource and bona fide researchers can apply to use the UK Biobank dataset by registering and applying at https://www.ukbiobank.ac.uk/enableyourresearch/apply-for-access. The data and analysis codes used for this study are going to be available on the UK Biobank website for registered researchers at the UK Biobank and an application fee.
