# Supplementary material for: Reevaluating the fraction of cancer attributable to excess weight: overcoming the hidden impact of prediagnostic weight loss
Source: Eur J Epidemiol. 2024 Sep 18;39(9):991–1003. doi: 10.1007/s10654-024-01146-0 (PMC11470860; doi:10.1007/s10654-024-01146-0)
Supplement: Supplementary file 1 — Supplementary Material 1 [file 10654_2024_1146_MOESM1_ESM.docx]

**Supplemental material**

**Reevaluating the fraction of cancer attributable to excess weight: overcoming the hidden impact of prediagnostic weight loss**

Fatemeh Safizadeh, Marko Mandic, Michael Hoffmeister, Hermann Brenner

**Supplemental Table 1.** List of cancer types (ICD-10) associated with excess weight considered in this analysis.

**Supplemental Table 2.** Prevalence of overweight and obesity in England, by survey year, age, and sex.

**Supplemental Table 3.** Hazard ratios (95% CIs) and population attributable fractions (95% CIs) for the incidence of overall, GI, and non-GI obesity-related cancers associated with overweight and obesity, obtained with inclusion of 0-3/>3-14 and 0-5/>5-14 follow-up time windows in the analyses.

**Supplemental Table 4.** Subgroup-specific hazard ratios (95% CIs) for the incidence of obesity-related gastrointestinal cancers, obtained with inclusion of various follow-up time windows after recruitment in the analyses.

**Supplemental Table 5.** Subgroup-specific hazard ratios (95% CIs) for the incidence of obesity-related non-gastrointestinal cancers, obtained with inclusion of various follow-up time windows after recruitment in the analyses.

**Supplemental Text:** Miettinen’s Population Attributable Fraction (PAF) formula modified for a multicategory risk factor.

**Correspondence to:**

Hermann Brenner

Division of Clinical Epidemiology and Aging Research, German Cancer Research Center

Im Neuenheimer Feld 581, D-69120 Heidelberg, Germany

Phone: +49 (0)6221 42 1301

Email: h.brenner@dkfz-Heidelberg.de

**Supplemental Table 1.** List of cancer types (ICD-10) associated with excess weight considered in this analysis.

| **Cancer type** | | **ICD-10 code** | **Incident cases^a^ [N]** |
| --- | --- | --- | --- |
| Gastro-intestinal | Esophagus | C15.0-C15.5, C15.8, C15.9 | 958 |
|  | Stomach (cardia) | C16.0 | 261 |
|  | Colorectum | C18.0- C18.9, C19, C20 | 5,410 |
|  | Liver | C22.0, C22.1, C22.3, C22.4, C22.7, C22.9 | 573 |
|  | Gall bladder | C23 | 109 |
|  | Pancreas | C25.0-C25.4, C25.7, C25.8, C25.9 | 1,160 |
|  | Total |  | 8,460 |
| Other | Breast (post-menopausal^b^) | C50.0-C50.6, C50.8, C50.9 | 6,984 |
|  | Endometrium | C54.1 | 1,360 |
|  | Kidney | C64 | 1,333 |
|  | Ovary | C56 | 892 |
|  | Meningioma | C70.0, C70.9 | 13 |
|  | Thyroid | C73 | 405 |
|  | Multiple myeloma | C90.0 | 788 |
|  | Total |  | 11,765 |
| Total |  |  | 20,218 |

^a^The addition of incident cancer cases for each cancer type exceeds the total number of cases due to multiple incident cancers diagnosed on the same date for a number of participants.

^b^Breast cancers were considered postmenopausal if either of the following conditions was met: Reported menopause or history of bilateral oophorectomy at baseline, or age ≥55 years at the time of breast cancer diagnosis.

**Supplemental Table 2.** Prevalence of overweight and obesity in England, by survey year, age, and sex (summarized from Health Survey for England – 2010 [1]).

| **BMI category (kg/m^2^)**^a^ | **Survey year** | | | | |  |
| --- | --- | --- | --- | --- | --- | --- |
|  | 2006 | 2007 | 2008 | 2009 | 2010 | Average |
|  | % | % | % | % | % | % |
| **Men** |  |  |  |  |  |  |
| **40-44** |  |  |  |  |  |  |
| Normal | 26.5 | 27.7 | 26.7 | 28.5 | 23.6 | 26.6 |
| Overweight | 48.1 | 46.5 | 46.5 | 49.8 | 48.6 | 47.9 |
| Obese | 25.0 | 24.8 | 26.1 | 21.2 | 27.6 | 24.9 |
|  |  |  |  |  |  |  |
| **Women** |  |  |  |  |  |  |
| **40-44** |  |  |  |  |  |  |
| Normal | 45.2 | 41.0 | 42.0 | 46.4 | 42.1 | 43.3 |
| Overweight | 30.1 | 34.6 | 31.2 | 29.1 | 30.0 | 31.0 |
| Obese | 23.7 | 23.6 | 25.2 | 24.2 | 26.1 | 24.6 |
|  |  |  |  |  |  |  |
| **Men** |  |  |  |  |  |  |
| **45-54** |  |  |  |  |  |  |
| Normal | 23.7 | 24.7 | 25.3 | 20.4 | 21.1 | 23.0 |
| Overweight | 48.1 | 40.3 | 43.7 | 45.2 | 44.0 | 44.3 |
| Obese | 28.1 | 34.5 | 30.8 | 34.1 | 34.5 | 32.4 |
|  |  |  |  |  |  |  |
| **Women** |  |  |  |  |  |  |
| **45-54** |  |  |  |  |  |  |
| Normal | 36.9 | 37.3 | 35.3 | 35.7 | 35.8 | 36.2 |
| Overweight | 35.2 | 31.8 | 35.1 | 36.3 | 33.7 | 34.4 |
| Obese | 27.2 | 30.0 | 28.9 | 26.5 | 30.2 | 28.6 |
|  |  |  |  |  |  |  |
| **Men** |  |  |  |  |  |  |
| **55-64** |  |  |  |  |  |  |
| Normal | 19.9 | 20.5 | 21.4 | 18.3 | 19.0 | 19.8 |
| Overweight | 46.6 | 48.4 | 44.3 | 49.2 | 44.0 | 46.5 |
| Obese | 33.0 | 30.9 | 33.9 | 32.2 | 36.5 | 33.3 |
|  |  |  |  |  |  |  |
| **Women** |  |  |  |  |  |  |
| **55-64** |  |  |  |  |  |  |
| Normal | 33.0 | 31.6 | 30.6 | 30.0 | 28.6 | 30.8 |
| Overweight | 35.7 | 36.8 | 37.7 | 39.3 | 38.8 | 37.7 |
| Obese | 30.2 | 31.0 | 31.0 | 28.9 | 31.8 | 30.6 |
|  |  |  |  |  |  |  |
| **Men** |  |  |  |  |  |  |
| **65-69** |  |  |  |  |  |  |
| Normal | 19.2 | 22.9 | 16.6 | 18.6 | 18.2 | 19.1 |
| Overweight | 49.4 | 48.7 | 49.9 | 51.8 | 53.3 | 50.6 |
| Obese | 31.1 | 28.4 | 33.0 | 29.6 | 28.1 | 30.0 |
|  |  |  |  |  |  |  |
| **Women** |  |  |  |  |  |  |
| **65-69** |  |  |  |  |  |  |
| Normal | 26.5 | 29.6 | 27.4 | 23.8 | 26.0 | 26.7 |
| Overweight | 37.5 | 37.4 | 38.5 | 44.3 | 37.2 | 39.0 |
| Obese | 34.7 | 31.8 | 33.3 | 31.1 | 36.6 | 33.5 |

^a^BMI (kg/m^2^) categories are defined as: normal (≥18.5-<25), overweight (≥25-<30), and obesity (≥30).

**Supplemental Table 3.** Hazard ratios (95% CIs) and population attributable fractions (95% CIs) for the incidence of overall, GI, and non-GI obesity-related cancers associated with overweight and obesity, obtained with inclusion of 0-3/>3-14 and 0-5/>5-14 follow-up time windows in the analyses.

| **Included Follow-up years** | **N cases** | **HR^a^ (95% CI)** | | **PAF**^b^**^1^**  **(95% CI)** | **PAF**^b^**^2^ (95% CI)** |
| --- | --- | --- | --- | --- | --- |
|  |  | **Overweight** | **Obesity** |  |  |
| **Overall obesity-related cancers** |  |  |  |  |  |
| **0-3** | 4,478 | 1.11 (1.04-1.20) | 1.25 (1.15-1.36) | 9.9 (-5.9-24.9) | 4.3(-2.5-10.7) |
| **>3-14** | 15,661 | 1.20 (1.15-1.25) | 1.49 (1.43-1.56) | 19.3 (10.5-27.2) | 8.4 (4.5-11.8) |
|  |  |  |  |  |  |
| **0-5** | 7,917 | 1.16 (1.10-1.22) | 1.34 (1.26-1.42) | 14.2 (2.3-24.9) | 6.1 (1.0-10.7) |
| **>5-14** | 12,222 | 1.19 (1.14-1.25) | 1.51 (1.44-1.59) | 19.3 (9.4-28.2) | 8.4 (4.1-12.2) |
|  |  |  |  |  |  |
| **Obesity-related GI cancers** |  |  |  |  |  |
| **0-3** | 1,790 | 1.08 (0.96-1.21) | 1.07 (0.93-1.22) | 5.8 (-21.0-27.9) | ------ |
| **>3-14** | 6,642 | 1.16 (1.09-1.24) | 1.39 (1.30-1.49) | 17.0 (4.3-28.3) | ------ |
|  |  |  |  |  |  |
| **0-5** | 3,195 | 1.10 (1.00-1.20) | 1.18 (1.06-1.30) | 10.0 (-9.1-23.8) | ------ |
| **>5-14** | 5,237 | 1.16 (1.08-1.25) | 1.39 (1.29-1.51) | 17.3 (3.1-30.0) | ------ |
|  |  |  |  |  |  |
| **Obesity-related non-GI cancers** |  |  |  |  |  |
| **0-3** | 2,689 | 1.13 (1.03-1.24) | 1.37 (1.23-1.51) | 14.5 (-0.8-27.3) | ------ |
| **>3-14** | 9,025 | 1.22 (1.16-1.28) | 1.57 (1.48-1.66) | 21.0 (12.0-29.0) | ------ |
|  |  |  |  |  |  |
| **0-5** | 4,725 | 1.20 (1.12-1.28) | 1.44 (1.33-1.55) | 17.6 (6.5-27.4) | ------ |
| **>5-14** | 6,989 | 1.21 (1.14-1.27) | 1.58 (1.49-1.69) | 20.9 (10.6-29.9) | ------ |

^a^Adjusted for age, sex, height, ethnicity, socio-economic deprivation, education, smoking status, pack-years of smoking, alcohol consumption, physical activity, fruit, vegetable, red meat and processed meat intake, hormone replacement therapy (women only), menopausal status (women only), history of bowel cancer screening, history of mammography (women only), and family history of breast and colorectal cancer.

^b^Age- and sex-weighted average percentage of cancer cases that is estimated to be attributable to overweight and obesity.

^1^PAFs of cancer cases associated with overweight and obesity when only obesity-related cancer cases were considered.

^2^PAFs of cancer cases associated with overweight and obesity as a proportion of total cancer.

Abbreviations: *BMI* Body Mass Index; *CI* Confidence Interval; *GI* Gastrointestinal; HR Hazard Ratio; *PAF* Population Attributable Fraction.

**Supplemental Table 4.** Subgroup-specific hazard ratios (95% CIs) for the incidence of obesity-related gastrointestinal cancers, obtained with inclusion of various follow-up time windows after recruitment in the analyses.

| **Subgroup** | **Follow-up years** | **N cases** | **HR^a^ (95% CI)** | |
| --- | --- | --- | --- | --- |
|  |  |  | **Overweight** | **Obesity** |
| **Age** |  |  |  |  |
| <60 | 0-4 | 803 | 1.06 (0.90-1.26) | 1.16 (0.95-1.42) |
|  | >4-14 | 2,139 | 1.19 (1.07-1.33) | 1.40 (1.24-1.58) |
|  | 0-14 | 2,942 | 1.15 (1.05-1.26) | 1.34 (1.20-1.48) |
| ≥60 | 0-4 | 1,684 | 1.08 (0.95-1.21) | 1.10 (0.95-1.26) |
|  | >4-14 | 3,806 | 1.17 (1.08-1.27) | 1.38 (1.26-1.52) |
|  | 0-14 | 5,490 | 1.15 (1.07-1.23) | 1.29 (1.19-1.39) |
| **Sex** |  |  |  |  |
| Male | 0-4 | 1,526 | 1.09 (0.96-1.25) | 1.24 (1.07-1.44) |
|  | >4-14 | 3,595 | 1.25 (1.14-1.37) | 1.60 (1.45-1.76) |
|  | 0-14 | 5,121 | 1.20 (1.11-1.29) | 1.48 (1.36-1.61) |
| Female | 0-4 | 961 | 1.04 (0.90-1.21) | 0.99 (0.83-1.18) |
|  | >4-14 | 2,350 | 1.09 (0.99-1.20) | 1.18 (1.05-1.32) |
|  | 0-14 | 3,311 | 1.07 (0.99-1.17) | 1.12 (1.02-1.23) |
| **Smoking status** |  |  |  |  |
| Non-smoker | 0-4 | 1,059 | 1.09 (0.94-1.26) | 1.15 (0.97-1.37) |
|  | >4-14 | 2,607 | 1.10 (0.99-1.21) | 1.35 (1.21-1.50) |
|  | 0-14 | 3,666 | 1.09 (1.01-1.18) | 1.30 (1.18-1.42) |
| Former smoker | 0-4 | 1,106 | 1.14 (0.97-1.33) | 1.18 (0.99-1.41) |
|  | >4-14 | 2,565 | 1.26 (1.13-1.41) | 1.50 (1.33-1.69) |
|  | 0-14 | 3,671 | 1.22 (1.12-1.34) | 1.39 (1.26-1.54) |
| Current smoker | 0-4 | 301 | 0.82 (0.63-1.06) | 0.94 (0.69-1.27) |
|  | >4-14 | 754 | 1.21 (1.02-1.44) | 1.33 (1.09-1.63) |
|  | 0-14 | 1,055 | 1.08 (0.93-1.25) | 1.19 (1.01-1.41) |
| **Diabetes** |  |  |  |  |
| No | 0-4 | 2,239 | 1.07 (0.96-1.18) | 1.11 (0.98-1.25) |
|  | >4-14 | 5,328 | 1.15 (1.07-1.23) | 1.32 (1.22-1.43) |
|  | 0-14 | 7,567 | 1.13 (1.06-1.20) | 1.25 (1.18-1.34) |
| Yes | 0-4 | 237 | 0.89 (0.57-1.39) | 0.86 (0.56-1.33) |
|  | >4-14 | 594 | 1.61 (1.12-2.31) | 1.88 (1.31-2.68) |
|  | 0-14 | 831 | 1.30 (0.98-1.71) | 1.45 (1.11-1.90) |

^a^Adjusted for age, sex, height, ethnicity, socio-economic deprivation, education, smoking status, pack-years of smoking, alcohol consumption, physical activity, fruit, vegetable, red meat and processed meat intake, hormone replacement therapy (women only), menopausal status (women only), history of bowel cancer screening, history of mammography (women only), and family history of breast and colorectal cancer.

Abbreviations: *BMI* Body Mass Index; *CI* Confidence Interval; *HR* Hazard Ratio.

**Supplemental Table 5.** Subgroup-specific hazard ratios (95% CIs) for the incidence of obesity-related non-gastrointestinal cancers, obtained with inclusion of various follow-up time windows after recruitment in the analyses.

| **Subgroup** | **Follow-up years** | **N cases** | **HR^a^ (95% CI)** | |
| --- | --- | --- | --- | --- |
|  |  |  | **Overweight** | **Obesity** |
| **Age** |  |  |  |  |
| <60 | 0-4 | 1,397 | 1.19 (1.04-1.35) | 1.36 (1.18-1.58) |
|  | >4-14 | 3,699 | 1.24 (1.14-1.34) | 1.60 (1.46-1.74) |
|  | 0-14 | 5,096 | 1.22 (1.14-1.31) | 1.52 (1.42-1.64) |
| ≥60 | 0-4 | 2,331 | 1.14 (1.03-1.25) | 1.41 (1.26-1.58) |
|  | >4-14 | 4,287 | 1.25 (1.16-1.35) | 1.59 (1.47-1.73) |
|  | 0-14 | 6,618 | 1.21 (1.14-1.28) | 1.53 (1.43-1.63) |
| **Sex** |  |  |  |  |
| Male | 0-4 | 389 | 1.09 (0.84-1.41) | 1.20 (0.90-1.61) |
|  | >4-14 | 1,076 | 1.31 (1.11-1.55) | 1.78 (1.49-2.13) |
|  | 0-14 | 1,465 | 1.25 (1.08-1.43) | 1.60 (1.38-1.87) |
| Female | 0-4 | 3,339 | 1.14 (1.05-1.24) | 1.41 (1.29-1.55) |
|  | >4-14 | 6,910 | 1.22 (1.15-1.29) | 1.57 (1.47-1.67) |
|  | 0-14 | 10,249 | 1.20 (1.14-1.25) | 1.51 (1.44-1.60) |
| **Smoking status** |  |  |  |  |
| Non-smoker | 0-4 | 2,078 | 1.13 (1.01-1.25) | 1.48 (1.32-1.66) |
|  | >4-14 | 4,507 | 1.21 (1.13-1.30) | 1.61 (1.49-1.74) |
|  | 0-14 | 6,585 | 1.18 (1.11-1.25) | 1.57 (1.47-1.67) |
| Former smoker | 0-4 | 1,320 | 1.18 (1.03-1.35) | 1.30 (1.17-1.43) |
|  | >4-14 | 2,723 | 1.18 (1.07-1.30) | 1.45 (1.33-1.58) |
|  | 0-14 | 4,043 | 1.18 (1.09-1.28) | 1.41 (1.29-1.54) |
| Current smoker | 0-4 | 308 | 1.06 (0.82-1.38) | 1.19 (0.88-1.62) |
|  | >4-14 | 725 | 1.60 (1.33-1.92) | 2.01 (1.64-2.46) |
|  | 0-14 | 1,033 | 1.40 (1.21-1.63) | 1.70 (1.44-2.01) |
| **Diabetes** |  |  |  |  |
| No | 0-4 | 3,471 | 1.13 (1.04-1.22) | 1.34 (1.22-1.47) |
|  | >4-14 | 7,534 | 1.23 (1.16-1.30) | 1.58 (1.49-1.68) |
|  | 0-14 | 11,005 | 1.20 (1.14-1.25) | 1.50 (1.42-1.58) |
| Yes | 0-4 | 239 | 1.37 (0.82-2.30) | 1.79 (1.10-2.93) |
|  | >4-14 | 418 | 1.31 (0.90-1.91) | 1.70 (1.18-2.43) |
|  | 0-14 | 657 | 1.32 (0.98-1.79) | 1.73 (1.30-2.31) |

^a^Adjusted for age, sex, height, ethnicity, socio-economic deprivation, education, smoking status, pack-years of smoking, alcohol consumption, physical activity, fruit, vegetable, red meat and processed meat intake, hormone replacement therapy (women only), menopausal status (women only), history of bowel cancer screening, history of mammography (women only), and family history of breast and colorectal cancer.

Abbreviations: *BMI* Body Mass Index; *CI* Confidence Interval; *HR* Hazard Ratio.

**Supplemental Text:**

Miettinen’s Population Attributable Fraction (PAF) formula modified for a multicategory risk factor:

$$PAF= \frac{\sum_{x=1}^{x=L} \pi\left( x \right)RR_{U}(x)\frac{RR_{C}\left( x \right)-1}{RR_{C}(x)}}{\pi\left( 0 \right)+\sum_{x=1}^{x=L} \pi\left( x \right)RR_{U}(x)}$$

Where x is the level of exposure and L the number of exposure categories, π is the prevalence of the risk factor, and π(0) represents the prevalence of the minimum risk exposure value (in our study normal body mass index (BMI): 18.5≤BMI<25) which is assumed to be equal to 0. In this formula, RR_U_ and RR_C_ represent the unadjusted and fully adjusted hazard ratios for the association between each category of the risk factor (in our study overweight (25≤BMI<30) and obesity (BMI≥30)) with the risk of cancer incidence, respectively.

# References

1. National Health Service (NHS). Health Survey for England - 2010, Trend Tables: Adult trend tables 2010. Available at: https://digital.nhs.uk/data-and-information/publications/statistical/health-survey-for-england/health-survey-for-england-2010-trend-tables accessed on 10/03/2023.
